# The Association Between Sleep and Home Accidents Among Preschool Children in Türkiye: A Case–Control Study

**DOI:** 10.3390/children13020288

**Published:** 2026-02-19

**Authors:** Fatma Durak, Özlem Tezol

**Affiliations:** Department of Pediatrics, Faculty of Medicine, Mersin University, 33110 Mersin, Türkiye; ozlemtezol@mersin.edu.tr

**Keywords:** home accident, preschool child, sleep problems

## Abstract

**Highlights:**

**What are the main findings?**

- This study describes the association between the sleep characteristics of both preschool children and their mothers, as primary caregivers, and the occurrence of home accidents among children.
- Longer nocturnal sleep duration in the child, shorter nocturnal sleep duration in the mother, and maternal daytime sleepiness significantly increase the risk of home accidents in preschool children.

**What are the implications of the main findings?**

- Campaigns to prevent childhood home accidents should emphasize the maintenance of optimal sleep duration for both children and caregivers, as well as the prevention of daytime sleepiness in caregivers.
- To reduce the frequency and severity of injuries associated with home accidents, greater focus must be placed on improving the sleep hygiene of both children and their mothers.

**Abstract:**

Background: Both home accidents and sleep problems are prevalent health issues among young children. This study aimed to investigate the association between the sleep characteristics of both preschool children and their mothers and the occurrence of home accidents among children. Methods: In this analytical cross-sectional study, the home accident group consisted of 90 children who presented to the Mersin University Hospital Pediatric Emergency Department due to home accidents. The control group comprised 90 healthy children, matched for age and sex with the home accident group. Sleep patterns of both children aged 12–72 months and their mothers, as primary caregivers, were evaluated through face-to-face interviews with the mothers. Results: Each one-hour increase in the child’s total nocturnal sleep duration increased the risk of being in the home accident group by 1.63 times (95% CI: 1.19–2.21, *p* = 0.002). Conversely, each one-hour increase in the mother’s total nocturnal sleep duration reduced the risk of child home accidents by a factor of 0.72 (95% CI: 0.58–0.91, *p* = 0.006). Maternal excessive daytime sleepiness increased the risk of home accidents in children by 11.35 times (95% CI: 2.38–54.26, *p* = 0.002). Conclusions: Preschool children who have had home accidents and their mothers should be evaluated for sleep problems. To reduce the frequency and severity of injuries associated with home accidents, greater focus must be placed on improving the sleep hygiene of both children and their mothers.

## 1. Introduction

Accidents occurring within the home or in adjacent areas such as gardens, courtyards, and garages are defined as home accidents. Due to their high prevalence and preventability, home accidents constitute a significant public health problem. Children are at high risk for home accidents because they lack the ability to protect themselves and possess an innate curiosity toward exploration and learning. Furthermore, children aged 0–6 years are frequently exposed to home accidents as they spend the vast majority of their time at home [1,2].

Globally, an estimated one million childhood deaths and 10 million pediatric injuries are attributed to home accidents annually. In five developing countries (Bangladesh, Colombia, Egypt, Malaysia, and Pakistan), 56.8% of childhood injuries originated from home accidents. In European countries, a correlation has been demonstrated between the home environment and the probability of accidents, as compared to transport or other locations, involving children under the age of five [3,4,5]. In Türkiye, 25% of all child deaths in 2024 were attributed to external injuries and poisoning. Between 2014 and 2017, injuries accounted for 4.1% of all deaths among Turkish children under five years of age; notably, in 53% of these injury cases, the incident occurred within the home or its immediate surroundings [6,7].

Injury resulting from home accidents is the most prevalent cause of preventable morbidity and mortality among children. Consequently, studies investigating the risk factors of home accidents in preschool children are essential for providing evidence-based insights for preventive interventions [8]. In developed countries, several child-related factors—including male gender, increasing age, advanced motor skills, birth order, attention-deficit hyperactivity disorder, and low psychosocial scores—have been positively associated with home accidents in preschool children. For example, later-born children run a greater risk of experiencing thermal injuries, fractures, and poisoning [9]. Parent-related factors include young age, poor maternal mental health, alcohol and substance use, low education level, and low socioeconomic status [9]. Additionally, household-related factors such as living in social rented housing, single-parent family structure, and the number of children in the home have also been linked to these incidents [9]. In developing countries, child’s age, gender, primary caregiver, birth order, number of siblings, social class, income status, and injury behavior score have a significant association with a history of accidental injury. For example, male children experience significantly higher rates of unintentional injury than females [3,10].

As sleep is considered an integral component of general health, sleep quality is recognized as one of the indicators of an individual’s injury risk. Sleep problems are associated with accidental injuries because they negatively impact psychological and cognitive states as well as physical performance [11]. In addition to sociodemographic and behavioral factors, insufficient sleep duration and a lack of daytime sleep have been reported as factors that may increase the risk of injury in the pediatric population [9,12]. Sleep problems in childhood can increase the risk of accidents, and accidents can also increase the risk of sleep disturbances. The majority of pediatric and adolescent patients self-report changes in sleep after injuries [13,14]. Pain, inflammation, and psychological stress from injuries frequently lead to sleep disturbances. Moreover, sleep disruptions can increase the risk of reinjury, creating a cycle that threatens children’s health [15]. These facts underscore the need to address sleep problems in routine screening and post-injury surveillance in children.

The existing literature supports the premise that sleep disturbances are more prevalent alongside increasing injury rates in preschool children and that an association exists between injury-prone behaviors and sleep disorders [11,16]. In contrast, there is a paucity of data regarding the relationship between the sleep characteristics of caregivers and pediatric injuries, indicating a clear need for studies investigating this link. Sleep deprivation in caregivers may be associated with childhood injuries as it impairs caregiver supervision by causing daytime cognitive dysfunction, instability in alertness, and negative mood states [17]. The objective of this study is to investigate the association between the sleep characteristics of both preschool children and their mothers, as primary caregivers, and the occurrence of home accidents among children.

## 2. Materials and Methods

### 2.1. Study Design and Study Sample

In this analytical cross-sectional study, the home accident group consisted of 90 children who presented to the Mersin University Hospital Pediatric Emergency Department due to home accidents between 15 April 2025, and 15 November 2025, along with their accompanying mothers. The control group comprised 90 healthy children, matched for age and sex with the home accident group, who presented to the General Pediatrics Outpatient Clinic for well-child visits, along with their accompanying mothers. Verbal assent was obtained from the children, and written informed consent was provided by their mothers. The Mersin University Ethics Committee approved the study (MEU 9 April 2025; 2025/379).

One mother–child pair was included in the control group for every 1 mother–child pair in the home accident group, providing a sample size with a 95% CI, 80% power, and 1.0 ratio of sample size using the ’OpenEpi calculator (Version 3.01).’ In the preliminary study, 17 child–mother dyads from each group were included between 15 April 2025 and 15 May 2025, and mean scores of The Tayside Children’s Sleep Questionnaire (18.06 ± 9.48 vs. 12.35 ± 8.55), Pittsburg Sleep Quality Index (6.94 ± 3.11 vs. 5.59 ± 3.26), and Epworth Sleepiness Scale (7.35 ± 6.25 vs. 4.59 ± 2.96) were compared between the groups. The minimum sample size was calculated as 88 subjects for the home accident group and 88 subjects for the control group by comparing the means (https://www.openepi.com/SampleSize/SSMean.htm, accessed on 6 December 2025).

Inclusion criteria for both the home accident and control groups were as follows: being aged 12–72 months, having the mother as the primary caregiver, and presenting to the hospital accompanied by the mother. Exclusion criteria for both groups included: a diagnosis of chronic disease (including neuromotor, genetic, or metabolic disorders) or chronic infection in the child, continuous medication use, a diagnosis of neuropsychiatric or behavioral disorders or sensory impairments, and a diagnosis of cognitive impairment, memory disorders, sensory impairments, and/or neuropsychiatric disorders in the mother. Additionally, children with a history of home accidents within the previous six months were excluded from the control group. Anthropometric measurements of the children were performed. Sociodemographic characteristics, identification of home accidents, and sleep patterns were evaluated through face-to-face interviews with the mothers. The interview data were collected by using a set of paper-based surveys. Since mothers take the primary role in childcare within the family, more reliable and accurate information about children could be obtained from mothers in Turkish society. Therefore, the present study was conducted with mothers and evaluated the sleep characteristics of both preschool children and their mothers as primary caregivers.

### 2.2. Assessment of Sociodemographic and Sleep Characteristics

Data regarding the children’s age, gender, enrollment in part-time preschool education (none, nursery, or kindergarten), perinatal characteristics, and duration of breastfeeding were recorded. The age and educational level of both mothers and fathers were obtained (categorized as primary school ≤8 years and high school or college >8 years). Maternal employment status (yes/no), presence of a chronic disease (yes/no), and tobacco and alcohol use (yes/no) were investigated. Additionally, the number of children in the household, family type, monthly income (income< expenses, income≥ expenses) and residential area (rural, urban/sub-urban) were recorded.

Mothers were asked if their children took daytime naps (yes/no). The following two questions were also asked to the mothers: (i) During the past month, what time has your child usually gone to bed at night? (ii) During the past month, what time has your child usually gotten up in the morning? The child’s total nocturnal sleep duration was calculated based on the mother’s report of the child’s bedtime and wake up time.

### 2.3. Characterization of Home Accident Features

All participants were queried regarding any prior history of home accidents (yes/no). For the home accident group, the specific type of accident that necessitated a visit to the pediatric emergency department was recorded.

### 2.4. Scale for Mother’s Identification of Safety Measures Against Home Accidents for Children of 0–6 Years Age Group

Maternal attitudes toward safety measures for the prevention of home accidents were assessed using the ‘Scale for Mother’s Identification of Safety Measures Against Home Accidents for Children of 0–6 Years Age Group.’ This five-point Likert-type scale consists of 40 items, including 34 positive and 6 negative statements (scored as 5: always, 4: often, 3: sometimes, 2: rarely, and 1: never). Each item is assigned a score between 1 and 5, with total scores ranging from a minimum of 40 to a maximum of 200. Higher scores indicate an increase in the measures taken by mothers to protect their children from home accidents. The Cronbach’s alpha value of the Turkish scale developed by Çınar and Görak is 0.82 [18].

### 2.5. The Tayside Children’s Sleep Questionnaire (TCSQ)

The Tayside Children’s Sleep Questionnaire (TCSQ) was utilized to assess sleep disturbances in the children. Developed as a five-point Likert-type scale, the TCSQ evaluates the sleep habits of children aged 1–5 years (0: never, 1: once or twice a month, 2: once or twice a week, 3: three to five nights a week, and 4: every night). The questionnaire consists of a total of 10 items, one of which is not included in the scoring. Higher total scores indicate a greater risk of sleep disturbances. A total score of eight or above suggests the presence of a sleep disorder; specifically, scores between 8 and 12 are considered low risk, 13–20 moderate risk, and 21–36 high risk. The Cronbach’s alpha value of the Turkish-language version of the TCSQ adapted by Arat and Ceylan is 0.81 [19,20].

### 2.6. Pittsburg Sleep Quality Index (PSQI)

The Pittsburgh Sleep Quality Index (PSQI) was used to evaluate the sleep quality of the mothers. The PSQI is a scale that assesses sleep quality and disturbances over the previous one-month period. The scale consists of 18 scored items categorized into seven components: subjective sleep quality, sleep latency, sleep duration, habitual sleep efficiency, sleep disturbances, use of sleep medication, and daytime dysfunction. Each component is scored on a scale of 0 to 3, and the sum of these seven components yields the total PSQI score, which ranges from 0 to 21. A total score greater than 5 indicates ‘poor sleep quality.’ The Cronbach’s alpha value of the Turkish-language version of the PSQI adapted by Ağargün et al. is 0.80 [21,22].

### 2.7. Epworth Sleepiness Scale (ESS)

Maternal sleepiness was assessed using the Epworth Sleepiness Scale (ESS). The ESS is a simple, self-report, 8-item scale that measures an individual’s general level of daytime sleepiness. It aims to evaluate the frequency of falling asleep or dozing (0: never, 1: slight chance, 2: moderate chance, 3: high chance) in eight different daily life situations: sitting and reading, watching television, sitting inactive in a public place, being a passenger in a car for an hour without a break, lying down to rest in the afternoon, sitting and talking to someone, sitting quietly after a lunch without alcohol, and in a car while stopped for a few minutes in traffic. A total score greater than 10 indicates ‘increased daytime sleepiness.’ The Cronbach’s alpha value of the Turkish-language version of the ESS adapted by Izci et al. is 0.86 [23,24].

### 2.8. Statistical Analysis

Data were analyzed using the SPSS 21 statistics program (IBM Corp., version 21.0. Armonk, NY, USA). The Kolmogorov–Smirnov test and histograms were used to test for normality of data. Median (IQR, 25th-75th percentile), mean ± standard deviation, and number (percentage) values were stated. Two parametric values were compared using Student’s t-test and two nonparametric values were compared using the Mann–Whitney U test. The chi-square test was used for categorical variables. Binary logistic regression was performed with ENTER method to assess the risk of home accident occurrence and included the independent variables with values of *p* < 0.2 in univariate analysis (age, gender, total nocturnal sleep time, a history of previous home accidents, maternal educational level, maternal occupation, maternal tobacco/cigarette use, paternal educational level, monthly income, and mother’s daytime sleepiness). Odds ratios (ORs) were calculated at a CI of 95%. All statistical assessments were two-sided, and statistical significance was considered at *p* < 0.05.

## 3. Results

### 3.1. General Characteristics

The study included 90 mother–child pairs in the home accident group and 90 in the control group. Table 1 shows the comparison of sociodemographic and anthropometric characteristics. In the home accident group, the median age of the children was 36 (24–54) months, while it was 35 (22–52) months in the control group (*p* = 0.819). In terms of gender, 55.6% of the home accident group and 53.3% of the control group were male (*p* = 0.765). No statistically significant differences were found between the groups regarding gestational age, mode of delivery, birth order, or breastfeeding duration (*p* = 0.672, *p* = 0.530, *p* = 0.232, and *p* = 0.856, respectively). Furthermore, 68.9% of children in the home accident group and 66.7% in the control group were not enrolled in preschool education (*p* = 0.280). Mean weight-for-age and height-for-age z-scores were similar between the groups (*p* = 0.088 and *p* = 0.955). Maternal and paternal ages did not differ significantly between groups (*p* = 0.745 and *p* = 0.720, respectively). However, the percentage of mothers with a primary school education level was significantly higher in the home accident group (33.3% vs. 14.4%, *p* = 0.003). There were no statistically significant differences in the frequencies of maternal chronic disease, employment status, tobacco use, or alcohol consumption (*p* > 0.05). Chronic diseases among mothers in both groups included asthma, diabetes mellitus, hypertension, and hypothyroidism. No significant differences were observed regarding paternal education level, number of children in the family, monthly income status, family type, or residential area (*p* > 0.05).

The median Scale for Mother’s Identification of Safety Measures Against Home Accidents score was 173.5 (169–177) in the home accident group and 173 (165–176) in the control group (*p* = 0.271) (Table 1).

### 3.2. Home Accident Characteristics

The types of accidents necessitating a visit to the emergency department in the home accident group, in order of percentage, were falls (47.8%), poisoning (12.2%), bumps/blunt trauma (12.2%), sharp object injuries (8.9%), foreign body ingestion (6.7%), animal attacks (5.6%), burns (4.4%), and drowning (2.2%). Regarding prior history, 32.2% (n = 29) of the home accident group and 22.2% (n = 20) of the control group had a history of previous home accidents (*p* = 0.132).

### 3.3. Sleep Characteristics

Sleep characteristics are presented in Table 2. There were no statistically significant differences between the children in the home accident and control groups regarding nocturnal bedtime and morning wake up times (*p* = 0.679 and *p* = 0.301, respectively). The median total nocturnal sleep duration was 10 (9–11) hours in the home accident group and 10 (9–10) hours in the control group (*p* = 0.005). The frequency and duration of daytime napping did not show a statistically significant difference between the groups (*p* = 0.745 and *p* = 0.324, respectively).

The median TCSQ score was 8.5 (6–18) in the home accident group and 11.5 (5–18) in the control group (*p* = 0.647). The percentage of children with a TCSQ score of 8 or higher—indicating the presence of sleep disturbances—was 56.7% in the home accident group and 63.3% in the control group (*p* = 0.361) (Table 2).

The median nocturnal sleep duration of mothers in the home accident group was statistically significantly shorter compared to those in the control group (7 h vs. 7.5 h, *p* = 0.028). The median PSQI score was 5 (3–7) in the home accident group and 5 (3–8) in the control group (*p* = 0.575). The percentage of mothers with a PSQI score above 5—indicating poor sleep quality—was 38.9% in the home accident group and 44.4% in the control group (*p* = 0.450) (Table 2).

The median ESS score was 5 (2–9) in the home accident group and 3.5 (1–6) in the control group (*p* = 0.003). The percentage of mothers with an ESS score above 10—indicating excessive daytime sleepiness—was 18.9% in the home accident group compared to 2.2% in the control group (*p* < 0.001) (Table 2).

### 3.4. Associated Factors for Home Accident

Each one-hour increase in the child’s total nocturnal sleep duration increased the risk of being in the home accident group by 1.63 times (95% CI: 1.19–2.21, *p* = 0.002). A prior history of home accidents [OR (95% CI) = 2.36 (1.05–5.30), *p* = 0.037] and a maternal education level of primary school or lower [OR (95% CI) = 3.71 (1.45–9.53), *p* = 0.006] were also found to increase the risk of home accidents. An income status where monthly income was greater than or equal to monthly expenses increased the risk of being in the home accident group by 2.34 times (95% CI: 1.09–5.01, *p* = 0.029). Conversely, each one-hour increase in the mother’s total nocturnal sleep duration reduced the risk of child home accidents by a factor of 0.72 (95% CI: 0.58–0.91, *p* = 0.006). Maternal excessive daytime sleepiness increased the risk of home accidents in children by 11.35 times (95% CI: 2.38–54.26, *p* = 0.002). Associations between home accidents and sociodemographic and sleep characteristics are shown in Table 3.

## 4. Discussion

Both home accidents and sleep problems are prevalent health issues among young children; both conditions directly impact the physical growth, as well as the cognitive and behavioral development, of infants and children [1,2]. The findings of the present study indicate that a one-hour increase in a child’s nocturnal sleep duration is associated with a higher risk of being categorized into the home accident group, whereas an increase in maternal nocturnal sleep duration serves as a protective factor. Furthermore, this study demonstrated that maternal excessive daytime sleepiness significantly elevates the risk of home accidents among preschool children.

There is limited data in the literature regarding the association between children’s sleep characteristics and accidental injuries. A prospective cohort study conducted in Canada (2012–2017) [25] investigated the relationship between sleep problems and injury occurrence in a population under 18 years of age [25]. In school-aged children (7–12 years), short sleep duration on weekdays increased the risk of injury by 2.1 times, while sleep debt (sleeping longer on weekends compared to weekdays) increased the risk by 1.9 times. However, in preschool children, no statistically significant association was identified between sleep characteristics and injury risk [25]. In a two-year follow-up study of children aged 18–24 months in Rochester, NY, it was observed that unintentional injuries were more frequent among children who did not have adequate sleep. Each one-point increase in the inadequate sleep score increased the number of unintentional injuries by 1.2. This finding remained significant even after controlling for maternal age, maternal educational level, and the child’s temperament [26]. Sleep problems in Italian children under two years of age led to a 1.3-fold increase in the risk of unintentional injuries [27]. In our sample of children aged 12–72 months, we did not find a significant association between sleep disturbances—as assessed by the TCSQ—and the risk of home accidents. However, we identified a link between the child’s total nocturnal sleep duration and accident risk. In populations aged 18 and older, it has been reported that both long and short sleep durations increase the risk of falls, with those achieving the recommended adult sleep duration (7 h) having the lowest incidence of falls. Specifically, sleep durations of both ≤6 h/day and ≥8 h/day have been associated with an increased incidence of falling 1–2 times per year in adults [28]. The U-shaped relationship between sleep duration and fall risk observed in adults may also be applicable to preschool children. In our study, the median (IQR) nocturnal sleep duration of the home accident group was longer than that of the control group; it was observed that each one-hour increase in nocturnal sleep duration—where the median was 10 h—increased the risk of home accidents by 1.63 times. On the other hand, it is well-established that a daily sleep duration of less than 8 h increases the risk of accidents in the preschool age group [29]. Since abnormal sleep durations, including excessive and insufficient sleep times, are associated with developmental, neurobehavioral and physiological problems such as poor cognition, decreased memory and attention, and metabolic disturbances [30,31], it can be hypothesized that long sleep and short sleep durations are both associated with higher rates of home accidental injury in preschool children. In fact, the greater the degree of deviation of sleep from the optimal sleep time, the lower the likelihood of achieving the restorative and protective benefits of sleep itself. Currently, there is no definitive information regarding the optimal sleep duration required to minimize injury risk, nor is it clear how many consecutive days of nocturnal sleep deprivation are necessary to elevate this risk [11]. In future studies, analyses conducted by categorizing preschool children according to their daily sleep duration may help determine the optimal sleep patterns that serve as a protective factor against home accidents.

Daytime napping may serve as a countermeasure against the adverse effects of sleep deprivation and has been associated with cognitive and physical health in children under the age of five [32]. When the relationship between daytime napping and accidents was examined, it was reported that napping reduced the risk of falls in both the 1–2 and 3–5-year age groups. Among children under five years of age, the frequency of daytime napping was found to be significantly lower in those who experienced falls compared to those who did not [29]. In contrast, we found the frequency of daytime napping to be similar between the home accident and control groups.

In the present study, we found no significant association between maternal overall sleep quality—as assessed by the PSQI—and the risk of childhood home accidents. Similarly, Chiu et al. reported that none of the components of the primary caregiver’s sleep quality (sleep duration, sleep disturbances, sleep latency, habitual sleep efficiency, daytime dysfunction, use of sleep medication, and overall sleep quality) influenced the risk of injury in Chinese children aged 0–4 years [17]. In contrast to Chiu et al. [17], we determined that each one-hour increase in the maternal sleep duration component of the PSQI reduced the risk of childhood home accidents by a factor of 0.72. Given that adults with sleep insufficiency are known to have a higher risk of injury compared to those without [33], there is a clear need for studies investigating the optimal maternal sleep duration required to protect both the mothers themselves and their young children from home accidents.

A significant association between accident-related injuries and daytime sleepiness has been demonstrated in school-aged children [34,35]. [36]. Daytime sleepiness increased the risk of accidental injury by 1.2 times in Chinese children [34], whereas the absence of daytime sleepiness reduced the injury risk by a factor of 0.8 in Iranian children [35]. Furthermore, abnormal daytime sleep has been shown to significantly increase the risk of injury in adolescents [36]. Additionally, sleepiness among mothers in the postpartum period increased the frequency of dropping their infants [37]. These findings point to a potential link between daytime sleepiness and accident risk. In our study, we demonstrated that maternal daytime sleepiness increased the risk of home accidents in preschool children by 11.4 times. Suboptimal sleep, which leads to daytime sleepiness, may impair effective and adequate parental supervision, thereby making young children more vulnerable to home accidents. We suggest that preventing daytime sleepiness in mothers may protect their children from accident-related injuries. Although it has been proposed that familial factors, such as neglect or inattentiveness, are the primary causes of most home accidents [38], it should be kept in mind that parental sleep deprivation may be the underlying factor predisposing families to such neglect and inattentiveness.

An integrative review reported that children from both low- and high-income families carry a similar risk for home accidents [39]. Likewise, some previous studies conducted in Türkiye found no difference in household income levels between children who experienced home accidents and those who did not [40,41]. On the other hand, in high-income countries, low household income has been identified as a predictor of accidental injuries among preschool children [9]. From a different perspective, it has been reported that the relationship between income level and the risk of home accidents in preschool children may vary depending on the type of accident [42]. In our study, a neutral or positive financial status (income ≥ expenses) was found to be a factor that increased the risk of home accidents compared to a negative financial status (income < expenses). Due to high hunger and poverty thresholds in Türkiye, inquiries about income levels in surveys are sensitive; therefore, we asked participants to categorize their income status rather than providing quantitative data. Taken together, the current literature and our findings suggest that the relationship between economic level and home accidents in young children warrants further investigation.

When examining the functional profiles of children who experienced unintentional injuries, their daily behaviors and executive function skills were evaluated; it was found that these children had lower scores in behavioral regulation, metacognition, and global executive function compared to those who had not experienced unintentional injuries [43]. Alongside these child-related functional factors, other persistent individual and environmental characteristics may predispose children to recurrent home accidents. Our finding, which demonstrates that a prior history of home accidents increases the risk by approximately 2.4 times, can be interpreted within this context.

The majority of previous studies have reported that the most frequent types of home accidents in children aged 0–6 years are falls, burns, bumps, drowning, and injuries involving sharp objects [3,44,45]. According to a single-center study, falling is also the most common type of home accident among Turkish children in the 0–6 age group [40]. In Türkiye, an increased risk of home accidents in this age group has been associated with advanced maternal age, unemployed mothers, and low parental education levels [38]. Consistent with these reports, we identified falling as the most frequent type of accident and found that a low maternal education level was associated with a 3.7-fold increase in the risk of home accidents in young children.

The vast majority of caregivers of young children who experienced home accidents stated that these incidents occurred due to a lack of supervision or unsafe environments and should not be attributed to fate [46]. While reinforcing this awareness through campaigns to prevent childhood home accidents, we recommend emphasizing the maintenance of optimal sleep duration for both children and caregivers, as well as the prevention of daytime sleepiness in caregivers. Preventive approaches focusing on risk factors for home accident-related injuries in children should prioritize improving maternal sleep hygiene alongside enhancing socioeconomic conditions and parental supervision. The fact that our findings emerged independently of the ‘Mother’s Identification of Safety Measures Against Home Accidents’ score is a significant strength of our study.

Our study has several limitations. First, the subjective nature of the sleep assessments is a limitation as they may be subject to recall and reporting bias. Second, due to the cross-sectional and single-center design of the study, a causal relationship between risk factors and home accidents cannot be established, and the findings cannot be generalized to the entire population of Türkiye. Third, we did not investigate certain factors previously associated with home accident risk. For example, the absence of a separate kitchen and inadequate indoor lighting have been shown to increase the risk of home accidents in children under five [47]; we did not evaluate such environmental factors. While single-center studies from various countries emphasize the importance of safe home environments in preventing accidents among preschool children [3,38], a meta-analysis found limited evidence that modifying the physical home environment by removing potential hazards reduces childhood injuries in low- and middle-income countries [1]. In this context, our lack of data on physical home environments may not constitute a severe limitation. Furthermore, behaviors such as smoking, alcohol consumption, excessive internet use, and insufficient sleep have been identified as factors increasing the risk of unintentional injuries, including home accidents [36]. Although alcohol and substance use, mental health status, and media usage among mothers of children under five have been associated with child home accidents in some studies [9,48], we found no association between maternal smoking or alcohol use and child home accident risk; however, maternal screen and internet usage characteristics were not queried.

## 5. Conclusions

Home accidents are the main cause of preventable debilities and death among children and are related to poorer sleep hygiene. Preschool children who have had home accidents and their mothers should be evaluated for sleep problems. To reduce the frequency and severity of injuries associated with home accidents, greater focus must be placed on improving the sleep hygiene of both children and their caregivers. Providing training for parents on creating a healthy sleep environment and providing sleep hygiene education to preschool children and their mothers will improve their sleep hygiene.

## Figures and Tables

**Table 1:** Comparison of sociodemographic and anthropometric characteristics and scores of Scale for Mother’s Identification of Safety Measures Against Home Accidents for Children of 0–6 Years Age Group.

	Home Accident Group (*n* = 90)	Control Group (*n* = 90)	*p*
Child			
Age, months	36 (24–54)	35 (22–52)	0.819 *
Gender, male	50 (55.6)	48 (53.3)	0.765 **
Gestational age, weeks	38 (37–39)	38 (37–39)	0.672 *
Delivery, cesarean	61 (67.8)	57 (63.3)	0.530 **
Birth order			0.232 **
First	46 (51.1)	38 (42.2)	
≥ Second	44 (48.9)	52 (57.8)	
Duration of breastfeeding, months	15 (6–22)	15 (6–24)	0.856 *
Receiving part-time preschool education			0.280 **
None	62 (68.9)	60 (66.7)	
Nursery	15 (16.7)	22 (24.4)	
Kindergarten	13 (14.4)	8 (8.9)	
z-Scores			
Weight for age	−0.10 (−0.56 to 0.61)	−0.43 (−0.95 to 0.61)	0.088 *
Height for age	0.01 (−0.86 to 0.73)	−0.03 (−0.93 to 0.73)	0.955 *
Parents			
Maternal age, years	32.5 ± 5.6	32.8 ± 5.8	0.745 ***
Maternal educational level			0.003 **
Primary school	30 (33.3)	13 (14.4)	
High school/college	60 (66.7)	77 (85.6)	
Maternal chronic disease, yes	10 (11.1)	16 (17.8)	0.203 **
Maternal occupation, employed	23 (25.6)	33 (36.7)	0.107 **
Maternal tobacco/cigarette use	11 (12.2)	18 (20.0)	0.156 **
Maternal alcohol use	3 (3.3)	5 (5.6)	0.720 **
Paternal age, years	36.4 ± 6.0	36.0 ± 6.7	0.720 ***
Paternal educational level			0.144 **
Primary school	23 (25.6)	15 (16.7)	
High school/college	67 (74.4)	75 (83.3)	
Family			
Number of children in the family	2 (1–2)	2 (1–2)	0.478 *
Family structure, nuclear	79 (87.8)	82 (91.1)	0.467 **
Monthly income,			0.124 **
Income < expense	29 (32.2)	39 (43.3)	
Income ≥ expense	61 (67.8)	31 (56.7)	
Settlement, urban/semi-urban	84 (93.3)	80 (88.9)	0.295 **
Scale for Mother’s Identification of Safety Measures Against Home Accidents for Children of 0–6 Years Age Group score	173.5 (169–177)	173 (165–176)	0.271 *

Bold values indicate statistical significance with a *p*-value less than 0.05. * Mann–Whitney U test; data are shown as median (IQR). ** Chi-square test; data are shown as numbers (percentage). *** Student’s t-test; data are shown as mean ± SD.

**Table 2:** Comparison of child and mother sleep characteristics.

	Home Accident Group (*n* = 90)	Control Group (*n* = 90)	*p*
Child			
Bedtime			0.679 **
20:00–21:00	25 (27.8)	20 (22.2)	
21:01–22.00	38 (42.2)	42 (46.7)	
After 22:00	27 (30.0)	28 (31.1)	
Morning wake up time			0.301 **
Before 07:00	29 (32.2)	34 (37.8)	
07:00–09:00	45 (50.0)	47 (52.2)	
After 09:00	16 (17.8)	9 (10.0)	
Total nocturnal sleep time, hours	10 (9–11)	10 (9–10)	0.005 *
Daytime napping, yes	62 (68.9)	64 (71.1)	0.745 **
Total daytime napping time, hours	2 (1.5–2)	2 (1.5–3)	0.324 *
The Tayside Children’s Sleep Questionnaire score	8.5 (6–18)	11.5 (5–18)	0.647 *
Score < 8, no risk for sleep disturbance	39 (43.3)	33 (36.7)	
Score 8–12, low risk for sleep disturbance	18 (20.0)	13 (14.4)	
Score 13–20, moderate risk for sleep disturbance	19 (21.1)	29 (32.2)	
Score 21–36, high risk for sleep disturbance	14 (15.6)	15 (16.7)	
The Tayside Children’s Sleep Questionnaire			
Score ≥ 8, sleep disturbance	51 (56.7)	57 (63.3)	0.361 **
Mother			
Duration of night sleep, hours (PSQI Duration)	7 (6–8)	7.5 (6–9)	0.028 *
Nocturnal sleep duration			0.091 **
≥ 8 h	28 (31.1)	42 (46.7)	
< 8 h and > 6	27 (30.0)	23 (25.6)	
≤ 6 h	35 (38.9)	25 (27.8)	
Pittsburg Sleep Quality Index score	5 (3–7)	5 (3–8)	0.575 *
Score > 5, poor sleep quality	35 (38.9)	40 (44.4)	0.450 **
Epworth Sleepiness Scale score	5 (2–9)	3.5 (1–6)	0.003 *
Score 0–5, lower normal daytime sleepiness	47 (52.2)	62 (68.9)	
Score 6–10, normal daytime sleepiness	26 (28.8)	26 (28.9)	
Score 11–12, mild daytime sleepiness	6 (6.7)	1 (1.1)	
Score 13–15, moderate daytime sleepiness	6 (6.7)	1 (1.1)	
Score 16–24, severe daytime sleepiness	5 (5.6)	0 (0)	
Epworth Sleepiness Scale			
Score > 10, daytime sleepiness	17 (18.9)	2 (2.2)	<0.001 **

PSQI, Pittsburg Sleep Quality Index. Bold values indicate statistical significance with a *p*-value less than 0.05. * Mann–Whitney U test; data are shown as median (IQR). ** Chi-square test; data are shown as numbers (percentage).

**Table 3:** Associations between child home accident and sociodemographic and sleep characteristics.

	B	*p*	Odds Ratio	95% CI for EXP(B)	
				Lower	Upper
Age, year	0.01	0.447	1.01	0.99	1.03
Gender, Male versus female	−0.08	0.819	0.92	0.46	1.86
Total nocturnal sleep time, hour	0.49	**0.002**	**1.63**	**1.19**	**2.21**
A prior history of home accidents, Presence versus absence	0.86	**0.037**	**2.36**	**1.05**	**5.30**
Maternal educational level, Primary school vs. high school/college	1.31	**0.006**	**3.71**	**1.45**	**9.53**
Maternal occupation Employed versus not employed	0.17	0.676	1.19	0.53	2.64
Maternal tobacco/cigarette use, Yes versus no	−0.91	0.066	0.40	0.15	1.06
Paternal educational level, Primary school vs. high school/college	0.53	0.301	1.69	0.62	4.59
Monthly income, Income ≥ expenses versus income < expenses	0.85	**0.029**	2.34	1.09	5.01
Mother’s nocturnal sleep duration, hour	−0.32	**0.006**	**0.72**	**0.58**	**0.91**
Mother’s daytime sleepiness (ESS score > 10), Presence versus absence	2.43	**0.002**	**11.35**	**2.38**	**54.26**
Constant	−4.06	0.010	0.02		

## Data Availability

The data that support the findings of this study are available from the corresponding author, Fatma Durak, upon reasonable request.

## Abbreviations

The following abbreviations are used in this manuscript:

TCSQ	The Tayside Children’s Sleep Questionnaire
PSQI	Pittsburg Sleep Quality Index
ESS	Epworth Sleepiness Scale
CI	Confidence interval
IQR	Interquartile range
